# Evidence of SARS-CoV-2 Related Coronaviruses Circulating in Sunda pangolins (*Manis javanica*) Confiscated From the Illegal Wildlife Trade in Viet Nam

**DOI:** 10.3389/fpubh.2022.826116

**Published:** 2022-03-09

**Authors:** Nguyen Thi Thanh Nga, Alice Latinne, Hoang Bich Thuy, Nguyen Van Long, Pham Thi Bich Ngoc, Nguyen Thi Lan Anh, Nguyen Van Thai, Tran Quang Phuong, Hoang Van Thai, Lam Kim Hai, Pham Thanh Long, Nguyen Thanh Phuong, Vo Van Hung, Le Tin Vinh Quang, Nguyen Thi Lan, Nguyen Thi Hoa, Christine K. Johnson, Jonna A. K. Mazet, Scott I. Roberton, Chris Walzer, Sarah H. Olson, Amanda E. Fine

**Affiliations:** ^1^Wildlife Conservation Society, Viet Nam Country Program, Ha Noi, Viet Nam; ^2^Wildlife Conservation Society, Global Conservation Program, New York City, NY, United States; ^3^Save Vietnam's Wildlife, Nho Quan, Viet Nam; ^4^Cuc Phuong National Park, Nho Quan, Viet Nam; ^5^Department of Animal Health, Ministry of Agricultural and Rural Development of Viet Nam, Ha Noi, Viet Nam; ^6^Regional Animal Health Office No. 6, Ho Chi Minh City, Viet Nam; ^7^Key Laboratory of Veterinary Biotechnology, Faculty of Veterinary Medicine, Viet Nam National University of Agriculture, Ha Noi, Viet Nam; ^8^School of Veterinary Medicine, One Health Institute, University of California, Davis, Davis, CA, United States; ^9^Research Institute of Wildlife Ecology, University of Veterinary Medicine, Vienna, Austria

**Keywords:** coronavirus, pangolin, SARS-CoV-2, one health, wildlife trade, trafficking, EID, spillover

## Abstract

Despite the discovery of several closely related viruses in bats, the direct evolutionary progenitor of SARS-CoV-2 has not yet been identified. In this study, we investigated potential animal sources of SARS-related coronaviruses using archived specimens from Sunda pangolins (*Manis javanica*) and Chinese pangolins (*Manis pentadactyla*) confiscated from the illegal wildlife trade, and from common palm civets (*Paradoxurus hermaphroditus*) raised on wildlife farms in Viet Nam. A total of 696 pangolin and civet specimens were screened for the presence of viral RNA from five zoonotic viral families and from Sarbecoviruses using primers specifically designed for pangolin coronaviruses. We also performed a curated data collection of media reports of wildlife confiscation events involving pangolins in Viet Nam between January 2016 and December 2020, to illustrate the global pangolin supply chain in the context of Viet Nam where the trade confiscated pangolins were sampled for this study. All specimens from pangolins and civets sampled along the wildlife supply chains between February 2017 and July 2018, in Viet Nam and tested with conventional PCR assays designed to detect flavivirus, paramyxovirus, filovirus, coronavirus, and orthomyxovirus RNA were negative. Civet samples were also negative for Sarbecoviruses, but 12 specimens from seven live pangolins confiscated in Hung Yen province, northern Viet Nam, in 2018 were positive for Sarbecoviruses. Our phylogenetic trees based on two fragments of the RdRp gene revealed that the Sarbecoviruses identified in these pangolins were closely related to pangolin coronaviruses detected in pangolins confiscated from the illegal wildlife trade in Yunnan and Guangxi provinces, China. Our curated data collection of media reports of wildlife confiscation events involving pangolins in Viet Nam between January 2016 and December 2020, reflected what is known about pangolin trafficking globally. Pangolins confiscated in Viet Nam were largely in transit, moving toward downstream consumers in China. Confiscations included pangolin scales sourced originally from Africa (and African species of pangolins), or pangolin carcasses and live pangolins native to Southeast Asia (predominately the Sunda pangolin) sourced from neighboring range countries and moving through Viet Nam toward provinces bordering China.

## Introduction

The role of animal intermediate hosts in the emergence of severe acute respiratory syndrome coronavirus 2 (SARS-CoV-2), and the role of the wildlife trade in facilitating the emergence of SARS-CoV-2 has not been determined. These are key questions for public health scientists, wildlife conservationists, and national policy makers working to identify the environments and circumstances under which viral spillover and transmission events occur in order to prevent future pandemics (1, 2). Specific questions about the origins of the virus, the context of early transmission events, and the potential role of intermediate animal hosts in the emergence of SARS-CoV-2, were raised as the first cases of the coronavirus disease 2019 (COVID-19) pandemic were being described (3) and the outbreak was declared a “global health concern” (4, 5). These questions remain pertinent 2 years later.

Following the identification of SARS-CoV-2 in the *Sarbecovirus* subgenus of the *Betacoronavirus* genus and *Coronaviridae* family (6), several coronaviruses (CoVs) phylogenetically closely related to SARS-CoV-2 were identified in horseshoe bats (*Rhinolophus* spp.) in Asia (7–16). The virus RaTG13, discovered in *R. affinis* in Yunnan province, China, in 2013 and three viruses identified in northern Laos, BANAL-52 (*R. malayanus*), BANAL-103 (*R. pusillus*) and BANAL-236 (*R. marshalli*) in 2020, are the closest relatives of SARS-CoV-2 known to date (9, 12). Importantly, the receptor binding domains of these viruses found in Laos efficiently bind to the human ACE2 receptor and are able to mediate entry into human cells in cell culture (12). More distant SARS-CoV-2 related CoVs have been discovered in China (*R. pusillus*, 2020; *R. malayanus*, 2019; *Rhinolophus* sp., 2018) (8, 10, 13), Japan (*R. cornutus*, 2013) (15), Thailand (*R. acuminatus*, 2020) (11), and Cambodia (*R. shameli*, 2010) (14), confirming that bats likely play a key role as evolutionary hosts for CoVs. Forests of Southeast Asia host a large diversity of horseshoe bats with hotspots of rhinolophid diversity identified in Viet Nam, Laos, and Southern China (10). The risk of rhinolophid-associated CoV emergence is not limited to China, and, likely, a large proportion of the bat CoV diversity in the region is yet to be discovered (12, 16).

Despite the identification of several closely related viruses in bats, the direct evolutionary progenitor of SARS-CoV-2 has not been determined. The search for a potential intermediate host is ongoing. Pangolins, and Sunda pangolins (*Manis javanica*) in particular, have been investigated as potential intermediate hosts of SARS-CoV-2 (11, 17–19). Authors of recent studies have suggested that pangolins naturally carry Sarbecoviruses, the viral subgenus containing SARS-CoV-1 and SARS-CoV-2, though the pangolin CoVs described to date are not considered direct evolutionary progenitors of SARS-CoV-2 (12, 20).

The *World Health Organization (WHO)-convened Global Study of Origins of SARS-CoV-2: China Part* examined possible pathways of emergence of SARS-CoV-2 through a qualitative risk assessment process (21). The authors considered the available scientific evidence at the time and concluded that direct zoonotic transmission was “possible to likely,” with potential introduction through an intermediate host followed by zoonotic transmission “likely to very likely.” In both cases, however, they encouraged studies of animal supply chains over a broader geographic area using a One Health approach. They noted the need to incorporate farmed wildlife, as well as an understanding of illegal trade routes/smuggling and wildlife ecology, to close current information gaps in understanding the risks of infectious disease emergence, spillover of SARS-like coronaviruses, and future pandemic threats.

In this study, we investigated potential animal sources of SARS-related CoVs using archived specimens from wildlife species collected in Viet Nam between February 2017 and July 2018, during USAID's Emerging Pandemic Threats Program PREDICT Project (7). Samples prioritized for testing included biological specimens from Sunda pangolins, Chinese pangolins (*Manis pentadactyla*), and common palm civets (*Paradoxurus hermaphroditus*). The pangolin specimens were collected in Viet Nam from live trafficked pangolins, representing wildlife moving through illegal wildlife supply chains. The civet specimens were collected from live common palm civets raised on registered farms in Viet Nam and part of the commercial wildlife farming industry. Specimens were screened for the presence of viral RNA from five zoonotic viral families (Coronaviridae, Filoviridae, Flaviviridae, Orthomyxoviridae, and Paramyxoviridae) and specifically for Sarbecoviruses. Data collected to characterize the human-wildlife interface along these wildlife supply chains at the time of sampling was cross referenced with concurrent wildlife farm registration data, pangolin rescue center records, and reports in the media of wildlife confiscation events in Viet Nam. Coordinating efforts to detect novel coronaviruses with research to characterize and identify the environments where spillover events may occur is urgently needed to inform risk mitigation strategies. This study takes the first steps in operationalizing a One Health approach which calls for looking at health, including emerging infectious diseases and potential pandemics, in the context of human, animal, and environment relationships (22).

## Materials and Methods

### Pangolin Sampling

The targeted sampling frame for the viral surveillance and specimen testing of pangolins in this study was pangolins trafficked through and confiscated in Viet Nam. The opportunity to collect specimens was only possible from those pangolins that were transferred to Save Vietnam's Wildlife (SVW) wildlife rescue center in Cuc Phuong National Park where specimen collection could be safely performed.

Sunda pangolins and Chinese pangolins were sampled February 2017 through July 2018. All pangolins sampled in this study were confiscated from the illegal wildlife trade in Viet Nam by Vietnamese law enforcement and forest protection agencies. They were all individual pangolins officially transferred to Cuc Phuong National Park in Ninh Binh province after confiscation. They were housed and maintained by SVW's wildlife rescue facility, located in the National Park, for care and assessment for potential release back into the wild. A protocol was established to sample the pangolins confiscated from the illegal wildlife trade within 1–2 weeks after their arrival at the center, while held in the SVW rescue center quarantine facility where they were housed individually and separated from any resident pangolins and other wildlife species. As pangolin sampling was initiated in February 2017, SVW had 10 individual pangolins in their quarantine facility awaiting placement or release. These pangolins were confiscated on 24 August 2015 (1 individual), 18 (1 individual) and 30 (1 individual) November 2016, 11 (1 individual), and 12 (5 individuals) December 2016, and 4 (1 individual) January 2017 (see Table 1). This “backlog” of previously confiscated pangolins in the rescue center was included in our sampling.

**Table 1:** Information from wildlife confiscation events associated with the pangolins sampled for testing in this study.

**Confiscation date**	**Confiscation province**	**Transportation route**	**Animals or products seized**	**No. pangolins sampled**
24-Aug-2015*^‡^	Ninh Binh	Da Nang-Ha Noi	7 pangolins, 5 turtles	1
18-Nov-2016*	Ha Noi	unknown	total unknown	1
30-Nov-2016*	Nghe An	Dak Nong-Ninh Binh	17 pangolins	1
11-Dec-2016*	Ninh Binh	Laos PDR-Ha Tinh-Nam Dinh	7 pangolins	1
12-Dec-2016*	Ninh Binh	Ha Tinh-Ha Noi	70 pangolins	5
4-Jan-17*	Da Nang	High land-Da Nang	4 pangolins	1
20-Jan-2017*	Thanh Hoa	unknown	28 rescued, total unknown	10
6-Apr-2017	Hoa Binh	Thanh Hoa-Ha Noi	118 pangolins	66
15-May-2017	Thanh Hoa	Nghe An-Quang Ninh	31 pangolins	1
4-Aug-2017	Ha Noi	unknown	33 rescued, total unknown	2
14-Sep-2017	Ha Noi	unknown	79 rescued, total unknown	1
20-Sep-2017	Ha Noi	unknown	8 rescued, total unknown	2
18-Nov-2017	Hung Yen	Ha Noi-Quang Ninh	112 pangolins	40
28-Jan-2018	Ca Mau	Transportation: by ship	114 pangolins, 300 kg of pangolin scales	10
4-May-2018	Quang Tri	Lao Bao international border gate (Lao PDR-Viet Nam)	15 pangolins	12
14-Jun-2018	Thanh Hoa	unknown	74 pangolins	54
25-Jun-2018	Ha Tinh	Lao PDR-Nghe An	7 pangolins, 1 dead pangolin, 26 turtles, 4 soft-shell turtles	2
9-Jul-2018**	Hung Yen	Lao PDR (suspected origin)	116 pangolins, 22 rescued	15
16-Jul-2018	Hai Phong	Quang Ninh-Hai Phong	2 pangolins	2
Pangolins sampled in 2017 and 2018 at Save Viet Nam's Wildlife Rescue Center in Cuc Phuong National Park with exact trade confiscation location unknown				19
**Total**				**246**

*Pangolins reported are live individuals unless indicated otherwise*.

* *The pangolins from these events were not sampled until February 2017*.

‡ *Event associated with the confiscation of 525 kg of animal intestines*.

** *Event associated with Sarbecovirus positive pangolins. Seven of the 15 pangolins sampled had positive Sarbecovirus results*.

Each sampled pangolin received a unique study identification code, SVW number, and SVW data record. These identifiers linked sampled pangolins to data available on wildlife confiscation events through media reports and the SVW wildlife rescue records. This allowed for additional data collection on specific confiscation events including rescue location, total number of pangolins confiscated, other wildlife species involved in the confiscation, and details of the illegal wildlife trade route recorded during the confiscation event.

**Figure 1:**
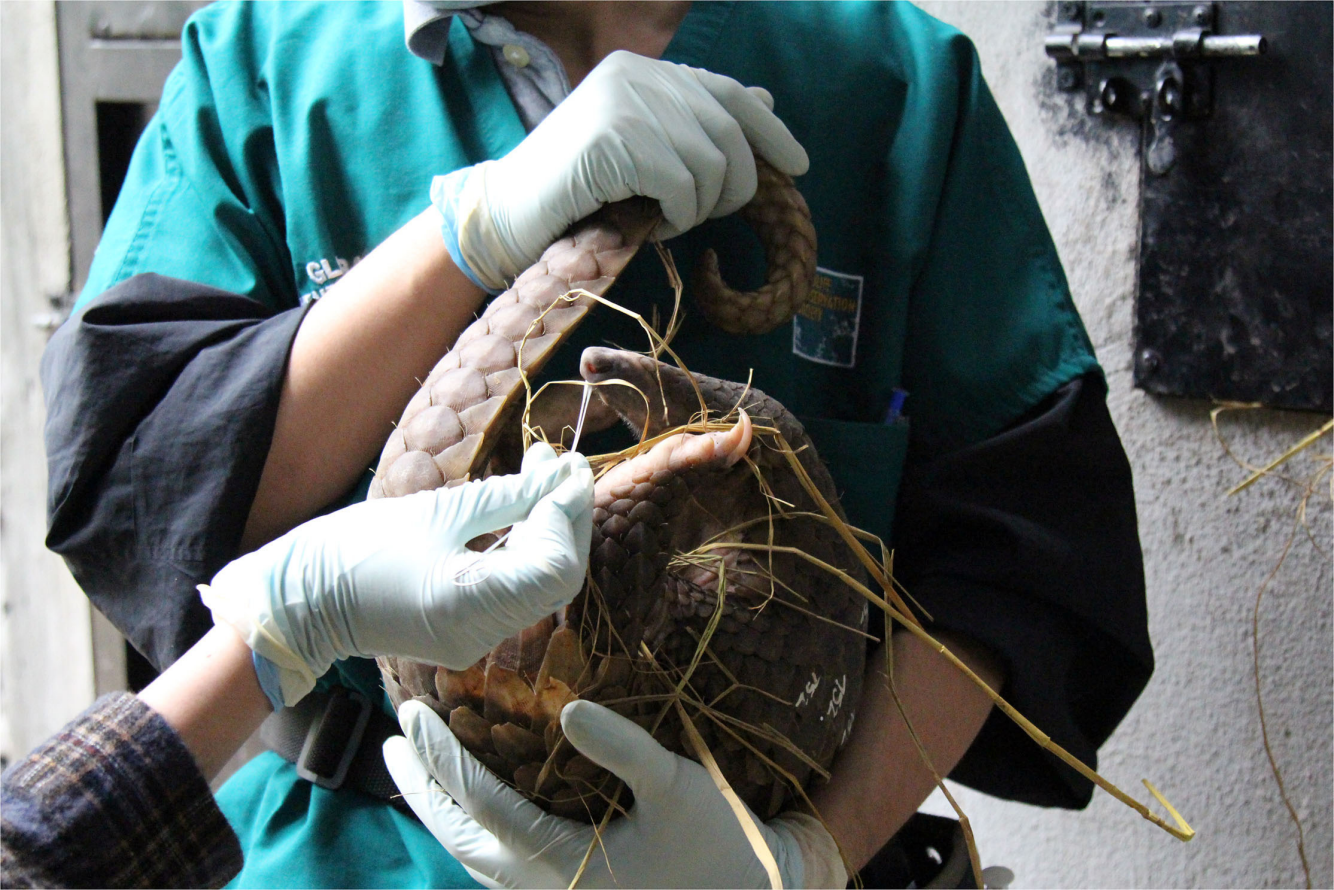
A young Sunda pangolin restrained manually for sample collection. It is possible to restrain small pangolins manually for sample collection if the procedure can be completed quickly in a quiet, low light area to limit stress on the pangolin. Photo courtesy of Wildlife Conservation Society, Viet Nam.

Both the pangolin and civet sampling protocols (see below) were approved by the University of California, Davis Institutional Animal Care and Use Committee (protocol number: 16048). Pangolin sampling and specimen collection were timed to coincide with the administration of SVW's routine animal intake health check. Young or weak pangolins were restrained manually during health checks (Figure 1). Larger adult pangolins, or those requiring procedures to treat wounds, were anesthetized with inhalant anesthetics (isoflurane) at a dose of 2–5% for initial box induction and 0.5–1.5% for maintenance *via* mask delivery. The sex, approximate age, weight, length, a photograph, and general condition of the pangolins sampled was recorded. The pangolins were identified to the species level by specialists familiar with the species and their distribution. Duplicate oral and rectal swabs were collected from the restrained pangolins using sterile, polyester-tipped swabs with a flexible metal shaft and placed in two labeled cryovials, one filled with 500 μL of TRIzol reagent (Invitrogen) and one filled with 500 μL Viral Transport Media (VTM) to maintain RNA integrity. Voided fresh fecal samples were also collected opportunistically from individual pangolins using sterile forceps with feces placed into two cryovials, one with 500 μL TRIzol, and one with 500 μL VTM. Specimens were stored in liquid nitrogen in the field before being transported to the Viet Nam National University of Agriculture where they were stored at −80°C until processing.

Oral swabs, rectal swabs, and/or fresh feces specimens were collected from individual pangolins during single handling and sampling events with the exception of five individual pangolins. These had oral swabs collected at one sampling event, and either rectal swabs or feces specimens collected during a second sampling event. The resampling date of these individuals is reflected in the Animal ID provided in Supplementary Material 1.

### Civet Sampling

Common palm civets were sampled August 2017 through July 2018, on seven registered civet farms or captive civet breeding facilities in Dong Nai province, Viet Nam. One of the farms selected raised civets for civet coffee and six of the farms selected raised civets for sale to restaurants or directly to consumers to be prepared and consumed as wild meat. All of the civet farms were either raising animals for sale and consumption as meat and/or for their use in the process of producing “civet coffee” which is made from partially digested coffee beans first fed to and then defecated by the civet. Both products are intended for human consumption as food. The sampling on civet farms was conducted in collaboration with the Dong Nai Forest Protection Department and the Dong Nai Sub-Department of Animal Health. During the sampling period, Dong Nai province had ~43 civet captive breeding facilities on record, representing 11.5% (43/373) of the civet farms registered in the 23 provinces of southern Viet Nam in 2017, and likely 3.7% (43/~1,170) nationwide (23). All civet farms selected had five or more civets registered on their farm. Among the seven civet farms, five of them (5/7 sites) were visited in the wet season (May–November), and four of them (4/7) were visited in the dry season (December–April). Sampling was repeated twice on two of the farms and three times on one of the farms.

**Figure 2:**
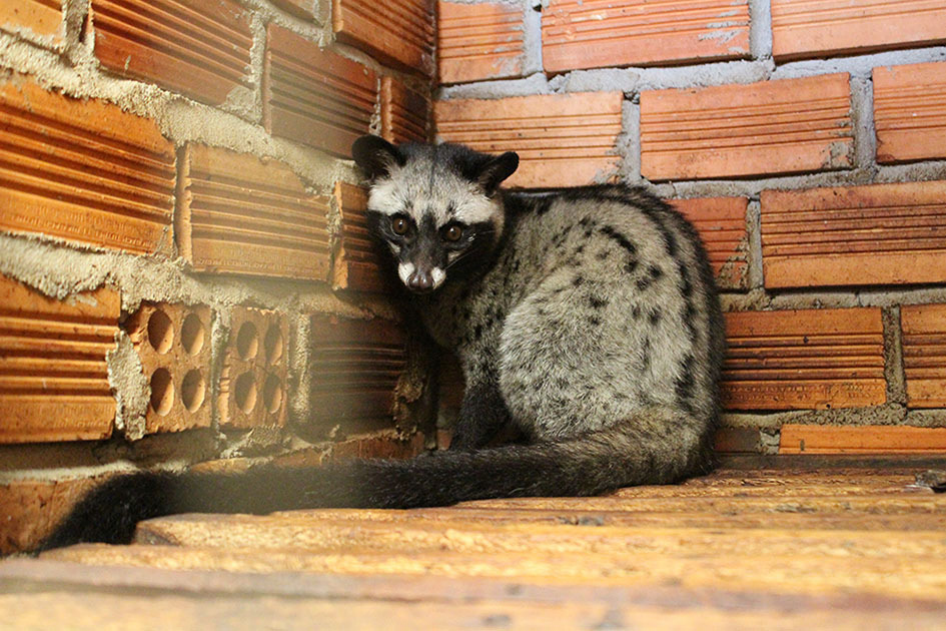
Common palm civet photographed in its individual cage on a wildlife farm in Viet Nam in 2017. If caged individually it is possible to design viral surveillance efforts around the collection of fresh voided fecal samples from individual animals. Photo courtesy of the Wildlife Conservation Society, Viet Nam.

Each sampled civet received a unique study identification code, and sex, approximate age, a photograph, and general condition of the animal were recorded. The species of civet was identified based on visual inspection and cross referenced with the farm registration data. The location of the civet farm, and the number of animals (both wild and domestic species) raised on the farm were recorded. No live animals were handled for specimen collection. Voided fresh fecal samples were collected from the floor of individually caged civets using sterile forceps with feces placed into two labeled cryovials, one with 500 μL TRIzol, and one with 500 μL VTM (Figure 2). Specimens were stored in liquid nitrogen in the field before being transported to the Regional Animal Health Laboratory Number 6 where they were stored at −80°C until processing.

### Specimen Testing

All specimens processed for screening in this study were collected into Trizol which inactivates any viruses. Specimens were screened in nationally certified BSL-2 laboratories at the Viet Nam National University of Agriculture (VNUA) in Ha Noi, and the Regional Animal Health Office No. 6 (RAHO6) in Ho Chi Minh City. The Sarbecovirus-specific screening of these specimens was performed at the RAHO6 laboratory which is approved by local authorities for SARS-CoV-2 testing.

#### Screening for Five Zoonotic Viral Families

All samples were screened for five priority viral families using a conventional PCR approach. RNA was extracted from samples with the RNA MiniPrep Kit (Sigma-Aldrich) and cDNA transcribed with the SuperScript III First Strand cDNA Synthesis System (Invitrogen). Broadly reactive PCR assays were then used to screen all samples for Coronaviridae, Filoviridae, Flaviviridae, Orthomyxoviridae, and Paramyxoviridae, following protocols outlined in Lee et al. (24) and Huong et al. (25). Assays developed by Watanabe et al. (26) and Quan et al. (27) [with a modification, as described in Lee et al. (24)] targeting two regions of the RNA-dependent RNA polymerase (RdRp) gene were used to screen samples for CoVs with consensus nested-PCRs. In each test batch, controls included distilled water (negative control) and a synthetic plasmid primer with binding sites for each assay (positive control).

#### Real-Time RT-PCR Assays Targeting SARS-CoV-2 and Sarbecoviruses

Pangolin and civet samples collected in 2017–2018 were tested using real-time reverse-transcriptase PCR (RT-PCR) assays targeting the E gene and RdRp gene developed by the Charité-Universitätsmedizin Berlin Institute of Virology (Germany) to detect SARS-CoV-2 (28). The E gene assay was designed as a first-line screening tool able to detect a wide range of Sarbecoviruses while the RdRp gene assay was a confirmatory assay targeting SARS-CoV-2 (28). Primers, probes, and PCR conditions used in this study were as described in Corman et al. (28). A conventional PCR was then performed on samples positive for the E gene real-time RT-PCR assay using the same primers. PCR products were sequenced after cloning to confirm virus identity.

#### Primer Design and PCR Assays Targeting Pangolin CoVs

Opportunities to pursue full genome sequencing to characterize the pangolin CoVs detected with the RT-PCR assays were explored immediately. However, national laboratories with capacity to perform full genome sequencing were not able to include the pangolin samples in their sequencing pipelines as resources were focused on clinical specimens from SARS-CoV-2 positive humans. In addition, expertise and resources to fully characterize unknown wildlife viruses remains limited in the country. It was also not possible to secure permission to export specimens outside Viet Nam. Conventional PCR approaches were therefore used to further describe the pangolin CoVs from Viet Nam.

Primers specific to pangolin Sarbecoviruses and targeting two regions of the RdRp gene corresponding to the conserved regions amplified in Watanabe et al. (26) and Quan et al. (27) were designed using the available pangolin CoV whole genomes as references (Supplementary Tables 1, 2). For each targeted region, three sets of primers were designed to be used individually in single-round PCRs or combined in nested PCRs (Table 2). Single-round PCRs using primer sets 1 or 2 and three primer combinations were tested for round 1 and 2 of nested PCRs: (A) primer set 1 (round 1) and set 2 (round 2), (B) set 1 (round 1) and set 3 (round 2), (C) set 2 (round 1) and set 3 (round 2). Some primer combinations were not tested for samples which gave positive results after single-round PCRs.

**Table 2:** Primers designed for this study and corresponding targeted regions.

	**Primer sequence**	Targeted region of reference genome GX-P5L*	**Amplicon size**
RdRp gene region 1 [corresponding to the region amplified in Watanabe et al. (26)]			
Primer set 1		15,237–15,691	455 bp
R1-Set1-Fw	5′-AAT-ATG-TTR-AAA-ACT-GTT-TAC-A-3′		
R1-Set1-Rev	5′-AAA-GCA-MAC-AAC-AGC-ATC-ATC-AGA-3′		
Primer set 2		15,286–15,678	393 bp
R1-Set2-Fw	5′-GGT-TGG-GAT-TAY-CCW-AAA-TGT-GA-3′		
R1-Set2-Rev	5′-GCA-TCA-TCA-GAR-AGT-ATC-ATC-ATT-3′		
Primer set 3		15,292–15,678	387 bp
R1-Set3-Fw	5′-GAT-TAY-CCW-AAA-TGT-GAY-AGA-GC-3′		
R1-Set2-Rev	5′-GCA-TCA-TCA-GAR-AGT-ATC-ATC-ATT-3′ (same as set 2)		
RdRp gene region 2 [corresponding to the region amplified in Quan et al. (27)]			
Primer set 1		18,361–18,799	439 bp
R2-Set1-Fw	5′-TTA-CAG-YTA-GGC-TTT-TCW-ACA-GGT-GT-3′		
R2-Set1-Rev	5′-CAT-GTG-CAT-TAC-CRT-GTA-CTT-G-3′		
Primer set 2		18,418–18,760	343 bp
R2-Set2-Fw	5′-YGT-TGA-YAC-AYC-TAA-TRM-WAC-AGA-KTT-YWC-3′		
R2-Set2-Rev	5′-RGT-CAT-GGT-TAC-TYT-GWA-RGT-TAC-CWG-TAA-A-3′		
Primer set 3		18,454–18,742	289 bp
R2-Set3-Fw	5′-RCC-ACC-ACC-TGG-WGA-CCA-RTT-3′		
R2-Set3-Rev	5′-TAA-AAC-CCC-ATT-GYT-GAA-CAT-C-3′		

* *Reference genome is the Pangolin CoV isolate PCoV_GX-P5L, GenBank accession number MT040335*.

PCRs were performed using 0.1 μl of Platinum Taq DNA polymerase, 0.5 μl of dNTPs (10 mM), 0.75 μl of MgCl_2_ (50 mM), 1 μl of each primer (10 μM) and 1 μl of template DNA in a 25 μl reaction. Cycle conditions included an initial denaturation of 2 min at 94°C, followed by 35 cycles of 20 s at 94°C, 30 s at 50°C, and 30 s at 72°C, and a final extension at 72°C for 7 min. The same conditions were used for PCR rounds 1 and 2 and all primer combinations. PCR products were visualized using 1.5% agarose gels. For each sample, if more than one of the tested primer combinations gave positive results, a single PCR product corresponding to the longest sequence length was selected for sequencing and TA cloning. Three clones from all samples were randomly selected and sequenced by Sanger dideoxy sequencing using the same primers as for amplification.

### Phylogenetic Analysis

Sequences from the two targeted regions of the RdRp gene were aligned in Geneious 9.0.2 (29) using the MUSCLE algorithm (30), and alignment was refined manually. Additional sequences of pangolin, bat, civet, mink, and human CoVs belonging to the Sarbecovirus lineage were added to our dataset (Supplementary Tables 1, 2). The Alphacoronavirus bat CoV BtKY72 was used as an outgroup as it was proven a suitable outgroup for Sarbecoviruses in recent phylogenetic studies (8, 10, 11, 17). Phylogenetic trees were inferred from each targeted region of the RdRp gene using PhyML 3.0 (31). Maximum likelihood inference was used with default parameters, as starting values and the substitution model was automatically selected. The starting tree was determined by BioNJ analysis of the datasets (32). Robustness of the tree was assessed by 1,000 bootstrap replicates.

### Pangolin Supply Chain Data Collection

In an effort to understand how our subset of sampled pangolins compared to the larger number of pangolins confiscated in Viet Nam during the period coinciding with this study, SVW's wildlife rescue records were combined with publicly available media reports of wildlife confiscation events involving pangolins in Viet Nam from January 2016 through December 2020. One pangolin included in this study was confiscated from the illegal wildlife trade in August 2015, but all others were confiscated from the illegal wildlife trade between November 2016 and July 2018. The 5-year period, January 2016 through December 2020, was selected for additional pangolin trade chain data collection to provide a broader picture of pangolin trafficking in Viet Nam during the period the individual pangolins included in this study were confiscated from the illegal wildlife trade.

The reports of wildlife confiscation events involving pangolins in Viet Nam were identified by searching a database maintained by the Wildlife Conservation Society (WCS) in Viet Nam. The database is populated by information gathered by using the Feedly tool to filter Vietnamese and English language news sites, blogs, Twitter, and newsletters on a daily basis for information on wildlife confiscation events in Viet Nam or involving Viet Nam as the origin, transit, or destination country. The results of the Feedly searches are sorted chronologically and each confiscation event reported is assessed to eliminate duplication. Each confiscation report in the database includes manually entered incident date and location, species involved, number of species, type and quantity of wildlife (live, dead/carcass, raw meat, scales, bone, processed product, etc.), type of transportation, origin, other trade nodes, or trade route if reported, and origin of shipment if the wildlife confiscation event involved a seizure of cargo at an international border point. The wildlife confiscation event database was searched for events involving pangolins, of known or unknown species, from the period of January 2016 through December 2020. Data were summarized to describe the location of confiscation events, border points or countries involved in the illegal trade, and type and quantity of pangolins and pangolin products confiscated. The confiscation events associated with pangolins sampled as part of this study were identified and mapped to display the geographic distribution of sampled pangolins from confiscation locations and relative number of pangolins sampled from these reported events.

## Results

### Samples Collected

**Figure 3:**
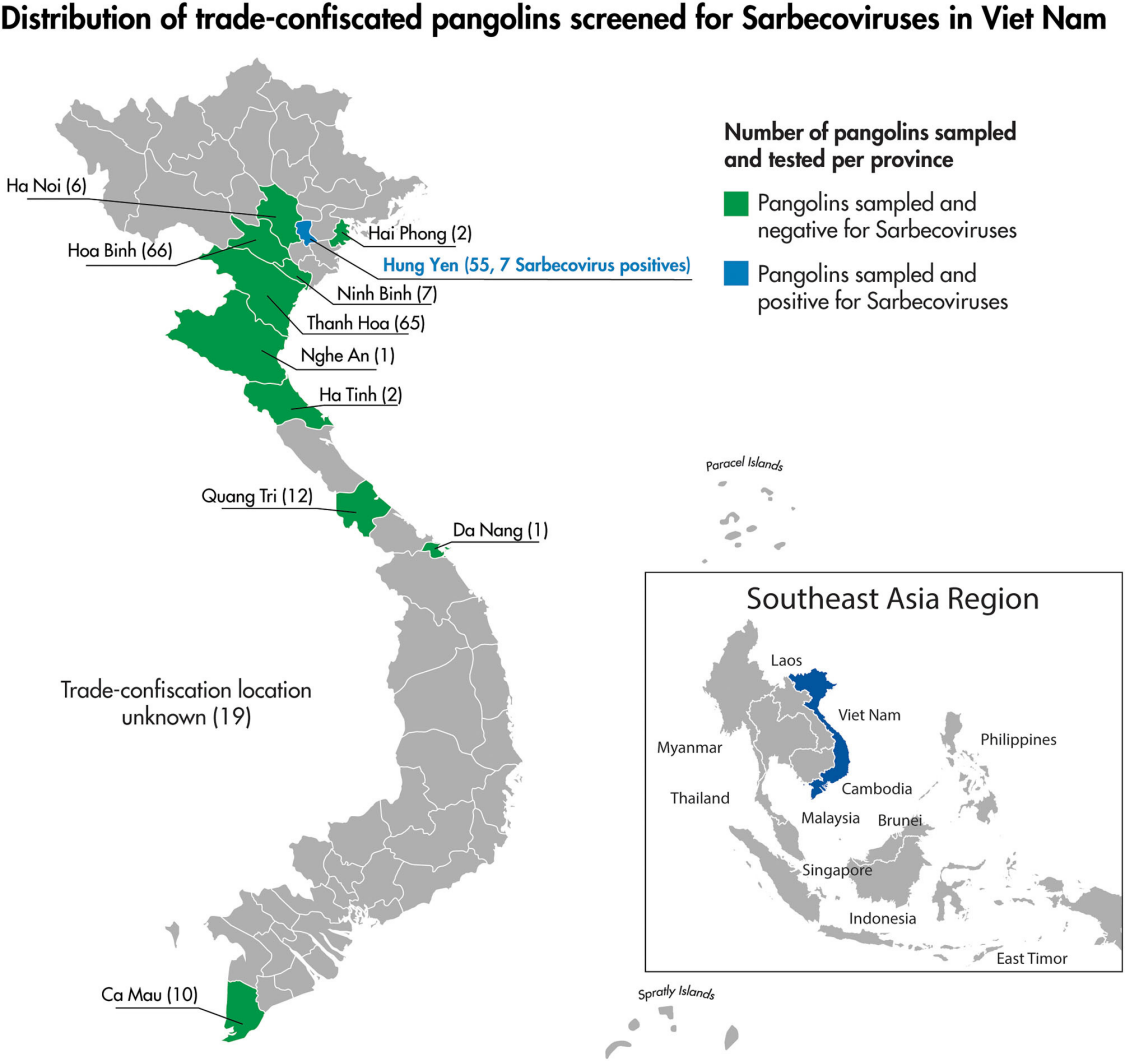
The location by province of pangolin confiscation events and the associated number of individual pangolins screened for Sarbecoviruses as part of this study.

We collected 397 specimens from 246 live pangolins confiscated from the illegal wildlife trade in Viet Nam. Pangolins sampled included three Chinese pangolins and 243 Sunda pangolins [Supplementary Material 1 and USAID Development Data Library (33)]. The majority of sampled pangolins (227/246) could be associated with specific wildlife confiscation events either reported in the media and entered into the wildlife confiscation database or described in the SVW wildlife rescue records. All of the confiscation events associated with sampled pangolins occurred between August 2015 and July 2018 (Table 1). These confiscation events occurred throughout the country in 10 provinces and the city of Ha Noi (Table 1; Figure 3).

Two hundred and ninety-nine fresh fecal samples were collected from the cages of individual civets at the seven targeted farm sites over 11 independent farm visits [Supplementary Material 1 and USAID Development Data Library (33)]. The civet coffee farm had the largest collection of civets (98 individuals at one sampling point), and the range of registered civets on the other farms was 9–30 individuals.

### Testing Results

All specimens from pangolins and civets sampled along the wildlife supply chains in Viet Nam and tested with conventional PCR assays designed to detect flavivirus, paramyxovirus, filovirus, CoV, and orthomyxovirus RNA were negative [Table 3, Supplementary Material 1 and USAID Development Data Library (33)].

**Table 3:** Results of pangolin and civet sample testing for five viral families using conventional PCR protocols and for SARS-COV-2 using the real-time reverse-transcriptase PCR (RT-PCR) assays targeting the E gene and RdRp gene (28).

**Point of sampling**	**Species name**	**No. individuals tested**	**No. specimens tested**	**No. individuals and specimens positive for Flavivirus, Paramyxovirus, Filovirus, Orthomyxovirus and Coronavirus**	**% Individuals and specimens positive for Sarbecoviruses - E-gene assay**	**% Individuals and specimens positive for Sarbecoviruses - RdRp-gene assay**
Wildlife farm	Common palm civet (*Paradoxurus hermaphroditus*)	299	299	0/299	0/299 (0%)	0/299 (0%)
Illegal wildlife trade	Chinese pangolin (*Manis pentadactyla*)	3	5	0/3 and 0/5	0/3 and 0/5 (0%)	0/3 and 0/5 (0%)
Illegal wildlife trade	Sunda pangolin (*Manis javanica*)	243*	392	0/243 and 0/392	7/243 (2.88%) and 12/392 (3.06%)	0/243 and 0/392 (0%)

* *Five individuals that were resampled had no positive viral findings. The resampling date of these individuals is reflected in the Animal ID provided in Supplementary Material 1*.

All civet samples tested using the E gene (first step) and RdRp gene (second step) RT-PCR assays (28) were negative [Table 3, Supplementary Material 1 and USAID Development Data Library (33)]. Among the pangolin samples, 12 specimens, including seven rectal swabs (7/7) and five oral swabs (5/7), from seven individual pangolins tested positive using the assay targeting the E gene, and those 12 specimens were negative when tested using the real-time RT-PCR assay targeting the RdRp gene (Table 3, Supplementary Table 3). The corresponding 65-bp E gene sequences were obtained from the 12 specimens using conventional PCR. All sequences obtained from pangolins confiscated in Viet Nam were identical or highly similar to sequences from SARS-CoV-2 and other Sarbecoviruses identified in bats and pangolins and were too short to discriminate among viral strains.

Rectal swab samples from the seven individual pangolins that tested positive for the E-gene assay were selected for PCR amplification using the new primer sets designed specifically for pangolin CoVs (Table 2). Among these seven samples, positive PCR results were obtained using primer set 1 for both RdRp regions for four samples (VNAA0207, VNAA0208, VNAA0218, VNAA0219). Samples VNAA0204, VNAA0226, VNAA0227 gave positive results when using various primer combinations in nested PCRs (Table 4). However, we were unable to amplify RdRp region 1 and region 2 for samples VNAA0226 and VNAA0204, respectively.

**Table 4:** Test results of single-round and nested PCRs performed on rectal swab samples from seven Sunda pangolins (*Manis javanica*) confiscated in Viet Nam.

	**RdRp gene region 1 (455 bp)**					**RdRp gene region 2 (439 bp)**				
	**Single-round PCRs**		**Nested PCRs**			**Single-round PCRs**		**Nested PCRs**		
**Animal ID**	**Set 1**	**Set 2**	**A**	**B**	**C**	**Set 1**	**Set 2**	**A**	**B**	**C**
VNAA0204	–	–	–	–	**+**	–	–	–	–	–
VNAA0207	**+**	+	NA	NA	NA	**+**	–	NA	NA	+
VNAA0208	**+**	+	NA	NA	NA	**+**	–	NA	NA	+
VNAA0218	**+**	+	NA	NA	NA	**+**	–	NA	NA	+
VNAA0219	**+**	+	NA	NA	NA	**+**	–	NA	NA	+
VNAA0226	–	–	–	–	–	–	–	**+**	+	+
VNAA0227	–	–	**+**	+	–	–	–	–	–	+

*PCR results highlighted in bold were selected for sequencing. Nested PCR A corresponds to primer set 1 (round 1) and set 2 (round 2), nested PCR B to set 1 (round 1), and set 3 (round 2), and nested PCR C to set 2 (round 1) and set 3 (round 2)*.

Most clone sequences of the RdRp gene region 1 obtained for samples VNAA0204, VNAA0207, VNAA0208, VNAA0218, VNAA0219, and VNAA0227 were identical. Slightly different sequences were also identified among the clones of samples VNAA0208 (VNAA0208/2 and VNAA0208/3) and VNAA0218 (VNAA0218/2 and VNAA0218/3). Identical sequences of the RdRp gene region 2 targeted in this study were also obtained for samples VNAA0207, VNAA0208, VNAA0218, VNAA0226, and VNAA0227. Slightly different sequences among the clones of samples were identified in VNAA0219 (VNAA0219/2 and VNAA0219/3), VNAA0207 (VNAA0207/2) and VNAA0218 (VNAA0218/2). All sequences were submitted to GenBank (Accession Numbers: OK510873-OK510892) (Supplementary Table 4).

**Figure 4:**
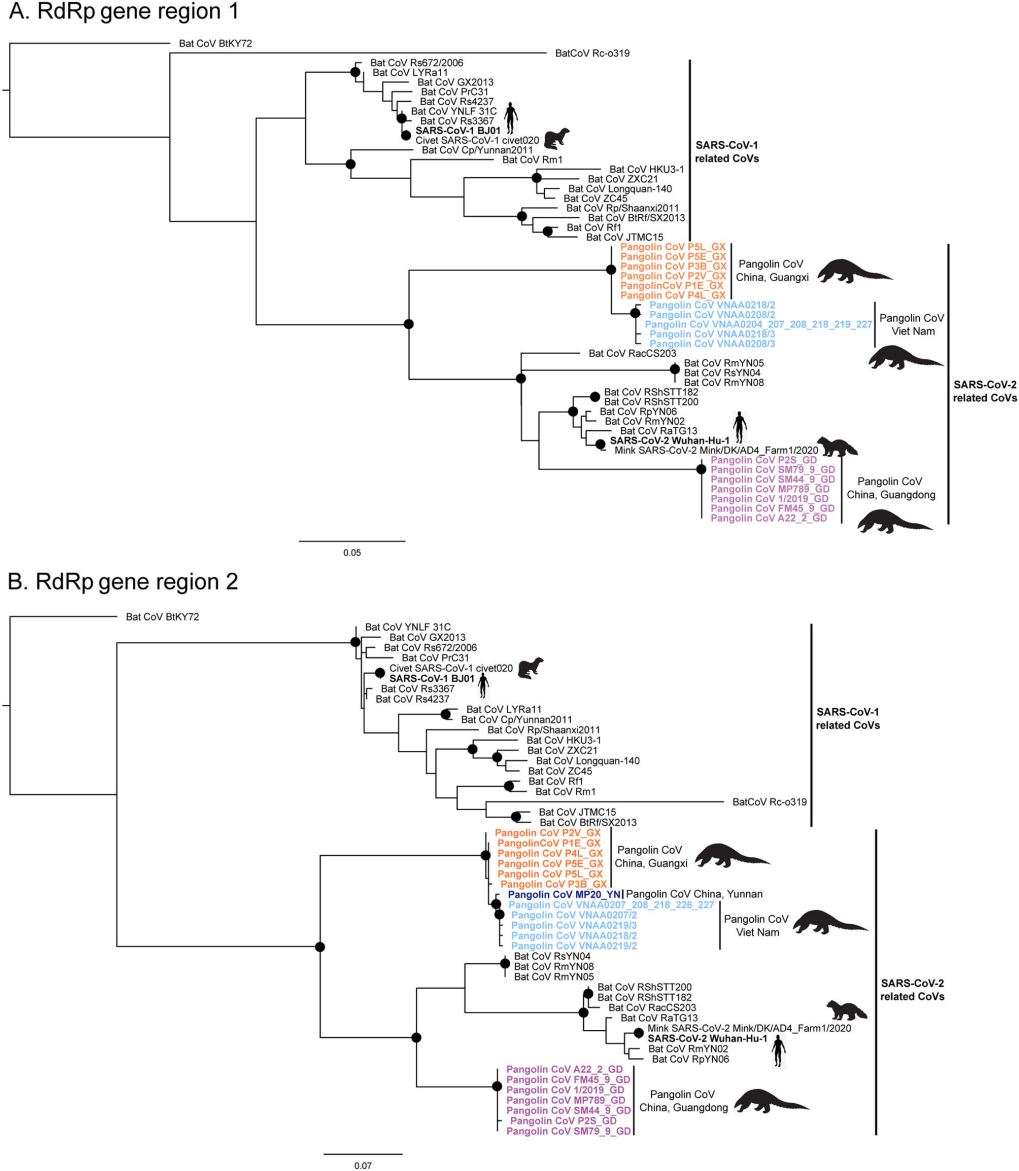
Maximum Likelihood phylogenetic trees (HKY85 +G+I) depicting phylogenetic relationships among Sarbecoviruses inferred from conserved RdRp gene region 1, 455 bp **(A)** and conserved RdRp gene region 2, 439 bp **(B)**. Pangolin CoV sequences from the Sunda pangolins confiscated from the illegal trade in Viet Nam are highlighted in dark blue. Pangolin CoV sequences from the Sunda pangolins confiscated from the illegal wildlife trade in the Chinese provinces of Guangdong (GD), Guangxi (GX), and Yunnan (YN) are highlighted in purple, orange, and dark blue, respectively. Well-supported nodes (bootstrap support > 75%) are indicated with a black dot.

The maximum likelihood phylogenetic trees for each of the two conserved RdRp gene regions targeted in this study reflect two main phylogenetic clades, one related to SARS-CoV-1 and one related to SARS-CoV-2, including pangolin and bat CoV sequences (Figure 4). The short CoV sequences detected in pangolins confiscated from the illegal wildlife trade in Viet Nam appear to cluster in a monophyletic subclade in both phylogenetic trees (Figure 4) and suggest they may be closely related and similar to CoVs detected in a Chinese pangolin confiscated from the illegal wildlife trade in Yunnan province in 2017, as well as from several Sunda pangolins confiscated from the illegal wildlife trade in Guangxi province, China, in 2017 (>99.2 and >98.7% sequence identity for the RdRp gene region 1 (455 bp) and RdRp gene region 2 (439 bp), respectively) (Table 5, Supplementary Table 1). This subclade including the Vietnamese and Chinese pangolin CoV sequences was first to diverge within the SARS-CoV-2 clade in both trees (Figure 4). CoV sequences detected in Sunda pangolins confiscated in Guangdong province, China, in 2019 were more divergent from those detected in Viet Nam (84–87% sequence identity for the two RdRp sequences used in this study) (Table 5) and belonged to another subclade more closely related to SARS-CoV-2 and SARS-CoV-2-related bat CoVs from China, Thailand, and Cambodia in both trees based on our RdRp sequences (Figure 4).

**Table 5:** Mean sequence identity and its range between Pangolin CoV sequences identified in Viet Nam and Pangolin CoV sequences identified in China (Yunnan, Guangxi, and Guangdong provinces) and SARS-CoV-2 in two conserved regions of the RdRp gene.

**Pangolin CoVs, Viet Nam**	**Pangolin CoVs, Yunnan, China**	**Pangolin CoVs, Guangxi, China**	**Pangolin CoVs, Guangdong, China**	**SARS-CoV-2**
RdRp gene region 1, 455 bp	NA	98.72 (98.68–98.90)	87.30 (87.25–87.45)	89.71 (89.67–89.89)
RdRp gene region 2, 439 bp	99.29 (99.23–99.49)	98.82 (98.46–99.09)	84.29 (84.25–84.47)	83.15 (83.10–83.33)

### Pangolin Trafficking in Viet Nam 2016–2020

The curated data collection of media reports of wildlife confiscation events involving pangolins in Viet Nam between January 2016 and December 2020, was performed to illustrate the global pangolin supply chain in the context of Viet Nam where the trade-confiscated pangolins were sampled for this study. The review was completed for the following three reasons: (1) to link the pangolins sampled at the SVW wildlife rescue center between February 2017 and July 2018 to specific confiscation events so the context of those confiscation events could be described in more detail, allowing us, for example, to begin to estimate the proportion of Viet Nam trade-confiscated live pangolins sampled during this study period; (2) to illustrate with information from specific confiscation events Viet Nam's role and trade node position in the global pangolin supply chain; and (3) to describe the confiscation events involving live pangolins in Viet Nam as a component of global pangolin trafficking. This allows us to view the trade-confiscated pangolins included in the viral surveillance study as part of the broader pangolin trade involving Viet Nam which is known to include pangolin species from Africa and Asia, transnational trade, and a range of products in addition to live pangolins.

**Figure 5:**
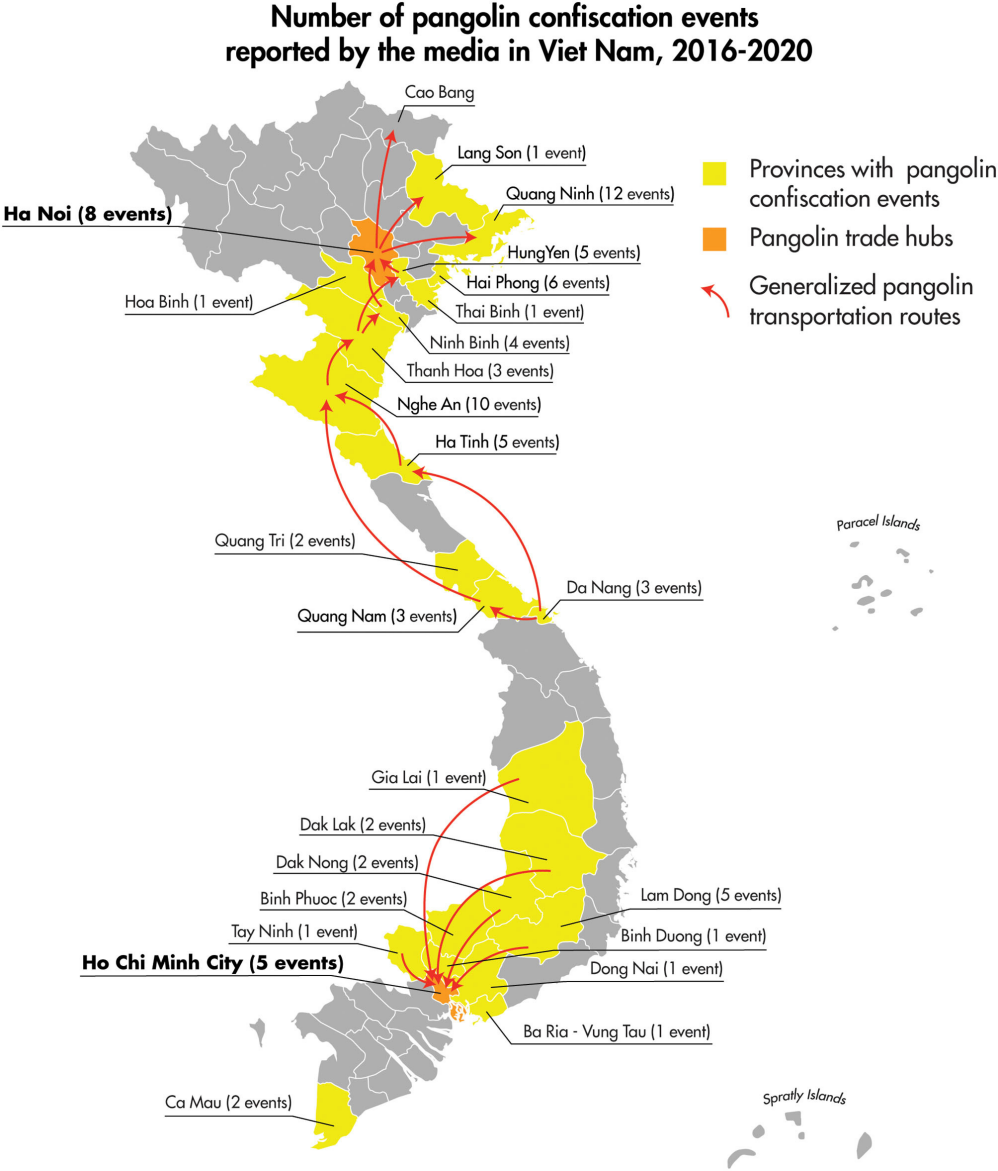
Location and number of pangolin confiscation events, and generalized depiction of domestic transport routes as reported in the public media for the period of January 2016–December 2020.

**Figure 6:**
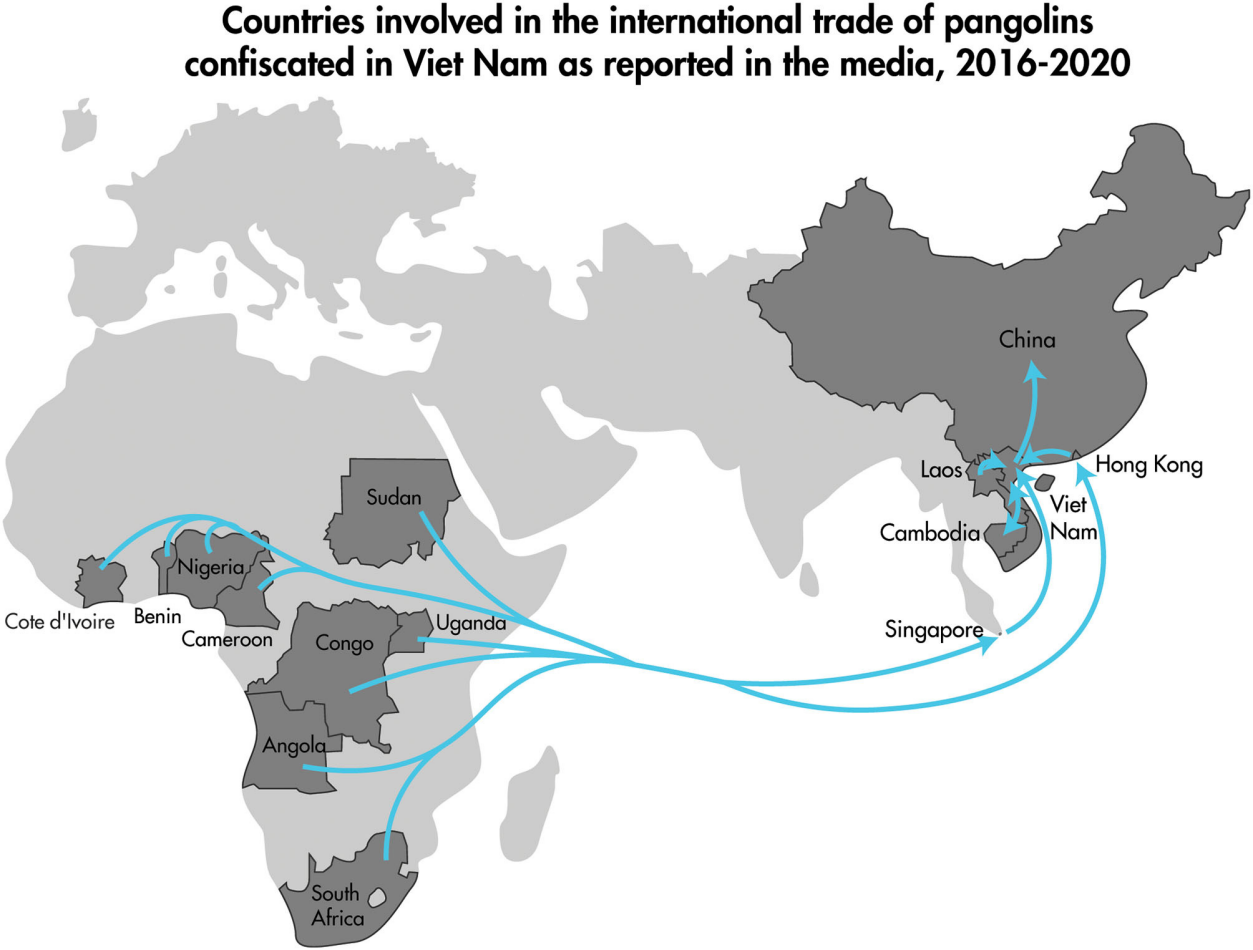
Map highlighting the countries identified in reports of trafficked pangolins confiscated in Viet Nam (labeled darker gray countries) covered in the media between January 2016–December 2020. The arrows depict the generalized direction of trade from Africa (mainly pangolin scales), and from Southeast Asia (mainly live pangolins and carcasses) to China.

The 2016–2020 curated data collection of media reports of pangolin confiscation events in Viet Nam identified 91 independent confiscation events carried out by law enforcement officials in 23 different provinces and the cities of Ha Noi and Ho Chi Minh. The confiscation events took place from the north to the south of the country and included coastal provinces as well as those bordering Laos, Cambodia, and China (Figure 5). Findings from the review of the 91 pangolin confiscation events reflected the trends in pangolin trafficking detailed in recent comprehensive reports on the pangolin trade, transport routes, and consumer demand for pangolins and pangolin products (34, 35). Pangolins and pangolin products were intercepted and confiscated by law enforcement agencies as they were transported by land (private vehicles and public transport), air, and sea. Countries identified in the reports as the origin of the shipments intercepted at international border points included Nigeria, Democratic Republic of the Congo, Cameroon, Angola, Ghana, Pakistan, Lao PDR, and Cambodia (Figure 6). Singapore and Lao PDR were also reported as transit countries (Figure 6). Confiscations involving African countries of origin were mostly shipments of pangolin scales transported by sea or air to the ports of Hai Phong, Ho Chi Minh City (Cat Lai), Da Nang (Tien Sa), Ba Ria Vung Tau (Cai Mep), or air shipments arriving at airports in Ha Noi (Noi Bai), and Ho Chi Minh City (Tan Son Nhat) with transit through Singapore and Hong Kong. Within Viet Nam and across land borders with Lao PDR, national roadways were the main means of transport with the majority of the confiscations involving live or dead pangolins. The general pattern of overland transport of pangolins within Viet Nam was from the central highland and southern provinces (Lam Dong, Dak Nong, Dak Lak, Binh Phuoc, and Dong Nai) toward Ho Chi Minh City, with the remaining movement of pangolins through transit provinces such as Thanh Hoa, Ninh Binh, and Hung Yen toward Ha Noi and on to the cross-border provinces with China of Quang Ninh, Lang Son, and Cao Bang (Figure 5).

The type and quantity of pangolins and pangolin products reported in the 91 confiscation events included a total of 1,342 live individuals (6,330 kg), 759 dead pangolins or pangolin carcasses (3,305 kg), and a total of 43,902 kg of pangolin scales. Further supporting the information on continental origin of pangolins from intercepted international shipments, the majority of pangolin scales were identified by law enforcement agencies as from African species of pangolins or unknown, and the majority of live and dead bodies of pangolins were identified as Asian pangolin species or unknown (Table 6). Just over one third (31/91 or 34%) of the confiscated pangolins were reported as accompanied by other wildlife or wildlife products. Live pangolins were confiscated with live rodents, live turtles, and other reptiles (snakes and crocodiles). Dead pangolins or pangolin carcasses were confiscated with carcasses of non-human primates and bear parts. Confiscated shipments of pangolin scales also contained elephant ivory and tortoise carapaces.

**Table 6:** Type and quantity of pangolins or pangolin products described in confiscation events reported in Viet Nam, Jan. 2016-Dec. 2020, and the species group (African, Asian, or Unknown) indicated.

**Continental**	**Carcasses**	**Live animals**	**Scales**
**origin**	**(individuals)**	**(individuals)**	**(kg)**
Africa	–	–	21,134
Asia	380	706	6,634
Unknown	379	636	16,135
**Total**	**759**	**1,342**	**43,903**

*Bold values along bottom row are total number or quantity for each column. Value at bottom of column 2 and 3 is total number of individuals, and value at bottom of column 4 is total quantify of scales in kg*.

## Discussion

In this study we investigated potential animal sources of SARS-related CoVs utilizing an existing set of wildlife specimens collected in Viet Nam from live pangolins (primarily the Sunda pangolin species native to Southeast Asia), and live common palm civets. Our investigation covered both illegal and legal wildlife supply chains represented by pangolins trafficked and confiscated from the wildlife trade and common palm civets raised on registered wildlife farms, respectively. Applying methods standardized for assessing risks of transmission of known pathogens along domestic animal supply chains to assessing the risks of novel virus spillover and amplification potential along wildlife supply chains is notoriously difficult. We strived to place virus surveillance findings in Sunda pangolins (and three Chinese pangolins) and common palm civets sampled in Viet Nam between February 2017 and July 2018 into context to facilitate an assessment of the risk for pathogen transmission and viral spillover along these supply chains. Although there are limitations when working with an existing set of specimens, by including data about specific nodes along the illegal and legal wildlife supply chains associated with wildlife at these sampling points we were able to begin to explore factors potentially increasing risk of spillover like geographic location, spatial movement, proximity, and potential contact between people, wildlife, and domestic animals. This context is what public health scientists, wildlife conservationists, and national policy makers are requesting to inform risk assessments and decisions about steps to take to respond to the increasingly voiced concerns that the expanding trade in live wildlife is among the most significant risk factors for the emergence of novel zoonotic-origin pathogens such as SARS-CoV-2 (1).

The majority of our pangolin viral surveillance data is from the Sunda pangolin, which is just one of the four species of pangolins that occur in Asia, and one of the eight species that occur globally (36). As this study confirms the Sunda pangolin is the species that is most commonly traded live through supply chains involving Viet Nam. Although live animal trade is the primary focus of investigations of the public health risks associated with the wildlife trade, we present additional data from wildlife confiscation reports. These data illustrate the full scope of pangolin trafficking in Viet Nam, which also includes the trade of African pangolin species almost exclusively in the form of pangolin scales. Research suggests that the Asian species of pangolins are capable of hosting *Betacoronaviruses* with published reports of SARS-CoVs identified in Sunda pangolins (17), Chinese pangolins (19), and a suspected human to pangolin transmission of SARS-CoV-2 in an Indian pangolin (37). Even less is known about the four species of African pangolins with literature knowledge gaps extending from the health and physiology of individuals to the conservation status and impacts of poaching on populations (36).

### Pangolin Sarbecovirus Screening

The identification of pangolin CoV sequences in specimens collected from live Sunda pangolins confiscated from the illegal wildlife trade in Viet Nam provides further evidence that pangolins host *Betacoronaviruses*. The pangolin CoVs described in this study were detected in seven Sunda pangolins associated with a wildlife confiscation event involving 116 live pangolins. These pangolins were seized by law enforcement authorities in Hung Yen province, bordering Viet Nam's capital city Ha Noi, in July 2018. Lao PDR was reported as the origin of these 116 live pangolins. The seven CoV positive Sunda pangolins represent 2.8% (*n* = 246) of the individual pangolins tested in this study, and the July 2018 confiscation event in Hung Yen province represents 5.3% (*n* = 19) of the confiscation events associated with testing for Sarbecoviruses.

Although full genome sequencing is required to fully characterize the pangolin CoVs detected in this study in 2018, and assess their zoonotic potential, our phylogenetic trees based on two short fragments of the RdRp gene suggested that these Vietnamese pangolin CoVs were most closely related to those detected in a Chinese pangolin confiscated from the illegal wildlife trade in Yunnan province in 2017 (19), and several Sunda pangolins confiscated from the illegal wildlife trade in Guangxi province, China, in 2017 (17). *In vitro* binding studies have shown that the receptor binding domain (RBD) of the Guangxi pangolin CoV was able to bind the human ACE2 receptor (11). Further investigation is now needed to assess the binding affinity and zoonotic potential of the pangolin CoVs detected in Viet Nam.

This study also revealed that neither of the two broadly reactive conventional PCR assays, nor the RT-PCR assay targeting the RdRp gene and specifically designed for SARS-CoV-2 used in this study, were able to successfully detect pangolin CoVs. Only the RT-PCR assay targeting the E gene designed by Corman et al. (28) and our own PCR assay based on pangolin-CoV specific primers were able to amplify short sequences of pangolin CoVs. These findings have important implications for further CoV surveillance efforts in pangolins and potentially other species. For example, the three PCR assays that were unsuccessful in this study in detecting pangolin CoVs were those used by Lee et al. (24) to conclude that pangolin samples collected in Malaysia were not infected by CoVs. Additional investigations using PCR primers specific to pangolin CoVs would therefore be needed to confirm these and other results.

### Civet Sarbecovirus Screening

Civets (Viverridae) are raised in captivity on wildlife farms in Viet Nam and traded commercially between farms and through wild meat restaurants. Common palm civet specimens collected from civets on wildlife farms in Dong Nai province were included in this study because the sale of live civets in markets in China was associated with the 2002–2003, and 2003–2004, outbreaks of severe acute respiratory syndrome (SARS) caused by what was then a newly emerged CoV (SARS-CoV-1) (38). Civets were one of three species of small carnivores in markets selling live wildlife in Southern China during the SARS outbreak found to be shedding SARS-CoV-1. In addition to the masked palm civet (*Paguma larvata*), SARS-CoV-1 was found in a racoon dog (*Nyctereutes procyonoides*), and a Chinese ferret badger (*Melogale moschata*) (39). We also know that a variety of wildlife species, including palm civets and raccoon dogs, were regularly sold in Wuhan markets in the months preceding the discovery of SARS-CoV-2 (40).

All of the civet specimens screened in this study for SARS-COV-2 using the RT-PCR assays targeting the E gene and RdRp gene were negative, as were the results from screening with broadly reactive conventional PCR assays designed to detect viruses in the Coronaviridae, Filoviridae, Flaviviridae, Orthomyxoviridae, and Paramyxoviridae families. Our sampling of the civet supply chain was limited to wildlife farms with the inclusion of an estimated 16% (7/43) of registered civet farms in one province (out of 63 provinces) in Viet Nam. Hence, the sample represents only a fraction of the civets raised in captivity and traded commercially in the country every year. Additionally, the number of civets on registered farms does not represent the true volume of the civet trade. An unknown number of civets in the Southeast Asia region enter the illegal trade when they are harvested from the wild to supply the demand of the illegal wildlife trade. Like other small carnivores hunted from inside and outside protected areas, they are sold on to wild meat restaurants, wildlife traders, or outlets selling traditional medicine or exotic pets (41). The common palm civet is also just one of 184 species of wildlife raised in captivity in Viet Nam (23), so although this is one of the largest viral surveillance efforts on wildlife farms in Southeast Asia it is limited in scope and to only one species in a complex supply chain of wildlife for human consumption as food.

### Sarbecovirus Screening Along Wildlife Supply Chains

The sampling of civets on wildlife farms, at the “source” rather than further along the civet supply chain, may explain the absence of viral RNA detection in this small cross-sectional sample. A comparative serological survey for SARS-CoV neutralizing antibodies in civets sampled on farms and in live animal markets in China during the SARS-CoV-1 outbreaks in June 2003 and January 2004, found the prevalence of exposure to be 79% (*n* = 18) in civets sampled in an animal market in Guangdong province as compared to a much lower prevalence of 10% (*n* = 75) in civets sampled from six farms across three Chinese provinces (42).

Studies of pathogens and the diversity of viruses found in pangolins are extremely rare which limits our ability to compare our results to other populations of pangolins. No CoVs were detected in 334 Sunda pangolins confiscated from smugglers or rescued from the wild between 2009 and 2019 in Malaysia (24). Subsequent Sarbecovirus-specific real-time RT-PCR screening using the RdRp assay (28) of the 334 Sunda pangolins in Malaysia also yielded negative results (33). Lee et al. (24) characterized the sampling of pangolins in Malaysia as “upstream” in the pangolin trade chain since habitat in Malaysia still supports significant numbers of Sunda pangolins and the individuals sampled most likely originated in Malaysia. In contrast, Lee et al. (24) characterized trade-confiscated pangolins sampled in China, where SARS-like CoVs were identified (17, 18, 20, 43) as “downstream” in pangolin supply chains. China is also outside the natural range of Sunda pangolins and wild Chinese pangolins in China are extremely rare, indicating pangolins confiscated in China are likely >1,000 km from their origin or “source sites” where they were hunted.

The main route for the illegal trade of Sunda pangolins in Southeast Asia has been documented as flowing along the Indonesia-Malaysia-Thailand-Lao-Viet Nam-China route. This is not always strictly linear, as there are points of supply and demand along the route and not all shipments will pass through each country, but it is considered the main illegal trade route for the Sunda pangolin (34, 44, 45). Our study therefore provides a “midstream” sampling point in the “upstream” to “downstream” pangolin supply chain described by Lee et al. (24), and this contributes additional insight into the role of the expanding trade in wildlife as a risk factor for the emergence of novel zoonotic-origin pathogens like SARS-CoV-2. Although there are very limited numbers of studies to which to compare our results, and sample sizes are small, the pangolin-CoV positivity rate of the live pangolins sampled in Viet Nam was 2.85% (*n* = 246). This value falls between the negative results from the pangolins sampled in Malaysia (0%) “upstream” in the supply chain, and the 6.98% (n=43) pangolin-CoV positivity rate for live Sunda pangolins sampled “downstream” in a rescue center in China (46). This progression in positivity rate aligns with Viet Nam's role as a major transit or “midstream” country in the trade of live pangolins and pangolin carcasses sourced from pangolin strongholds in other Southeast Asian countries. This apparent amplification of risk of CoV infection, measured by increasing virus detection rates, from source sites to large markets to urban consumption nodes along wildlife supply chains was also documented by studying the trade in live rodents for human consumption in the Mekong Delta Region of Viet Nam (25).

A recent study has also reported serological evidence of SARS-CoV infection in Sunda pangolins confiscated from the illegal wildlife trade in Thailand in 2020, with the serum from 1 of 10 pangolins tested showing a strong positive virus neutralization antibodies reading to a SARS-CoV-2 surrogate virus neutralization test (sVNT) (11). The researchers also reported historic data from pangolin sera collected in China in May 2003, in which one of seven serum samples was positive for SARS-CoV by competition ELISA and VNT (11). These data further support evidence that pangolins are susceptible to infection with SARS-like CoVs first reported in pangolins sampled “downstream” in the supply chain in China (17, 18, 20). The presence of antibodies to SARS-CoV in 10% of live pangolins screened in Thailand in 2020, and evidence of antibodies to SARS-CoV in a pangolin screened in 2003, indicates that pangolin infections with CoVs have likely been missed and are therefore significantly underreported due to a combination of under sampling and the viral discovery approaches used to date.

In this study we report on the detection of pangolin CoVs in seven Sunda pangolins out of a group of 15 that were sampled after being rescued from a wildlife confiscation event involving 116 individual pangolins. Since Sunda pangolins are solitary mammals in the wild, we assume that the high prevalence of pangolin CoV detection in this group (~50% of the individuals sampled) is the result of contact between pangolins that occurred once they entered the illegal wildlife supply chain. At the time of sampling none of the seven CoV-positive pangolins exhibited any obvious signs of systemic illness, beyond the typical skin abrasions and wounds around the head and feet often observed in trafficked pangolins. On-going transmission of the pangolin CoV among animals at the rescue center cannot be ruled out. However, SVW like many other wildlife rescue and rehabilitation centers around the world, have protocols and mitigation measures in place to reduce the risk of transmission of infectious agents among animals in their facilities or between animals and humans working at the rescue centers. International guidelines referenced and followed to put facility-specific protocols and contingency plans in place include the IUCN-OIE Manual for procedures for wildlife disease risk analysis (47) and other guidelines covering occupational health and safety in wildlife facilities.

It is not possible to apply methodologies used to determine virus prevalence along domestic animal supply chains to the wildlife trade, including in Viet Nam. Because the trade of many wildlife species is largely unregulated and often illegal, there are generally no defined points along the supply chain for health inspection or sample collection for disease screening. In this study we reviewed additional sources of data to put this opportunistic sample of specimens collected from trade-confiscated pangolins and farmed common palm civets into context. A publicly available national database of wildlife farms or wildlife farm-specific agriculture sector economic data does not exist. The actual number of civets moving through unregistered facilities and unregulated markets is unknown. It is, however, important to note that although the small sample size relative to the scale of the trade may have limited the opportunity to quantify risk and detect viral RNA from the viral families targeted in this study using conventional PCR assays, the collection of 299 civet specimens and 397 pangolin specimens represents one of the largest studies of pangolin and civet sample screening in the literature (17–19, 39, 42, 46).

### Wildlife Supply Chains and Regulation of Wildlife Trade

The curated data collection of media reports of wildlife confiscation events involving pangolins in Viet Nam allowed us to link the majority of pangolins sampled for this study to specific wildlife confiscation events. Wildlife confiscation events in Viet Nam often included a mix of taxa (non-human primates, reptiles, birds) and multiple pangolin confiscation events sampled in this study also involved other live wildlife and in one case domestic animal intestines which are traded outside slaughterhouse inspection systems (Table 1). In addition the review highlights the transnational nature of the wildlife trade, and also the transcontinental movement of wildlife and wildlife products as illustrated by the trade in pangolins and pangolin scales (Figure 6) supporting long held concerns that the wildlife trade, moving wildlife species out of their natural habitats and into human dominated landscapes and large urban centers, poses a serious and increasing risk of initiating epidemics from emergent pathogens in human populations (48–51).

Regulations based on protecting species from extinction have not been sufficient to end the trade in pangolins. All species of Asian pangolins, including the Sunda and Chinese pangolins are considered Endangered or Critically Endangered by the International Union for Conservation of Nature (IUCN) across their geographic range and all species of pangolins have been listed as CITES (the Convention on International Trade in Endangered Species of Wild Fauna and Flora) Appendix I since 2017, which prohibits all international trade. However, as the data from this study further confirms, large-scale poaching and trafficking of pangolins continues, fueled by a demand for their meat and scales in Southern China and to a lesser degree in other Southeast Asian countries (52–55). From the perspective of virus transmission, spillover risk, and spread, it is important to note that pangolin trafficking is often characterized by the transport of large groups of individuals that are housed together and often with other species of live wildlife, as this study confirms.

Current recommendations on regulation of live wildlife trade are narrowly focused on open markets and do not address the much longer wildlife supply chains and trade of both legally and illegally sourced wildlife. In April 2021 the WHO along with the United Nations Environmental Programme (UNEP) and the International Organization for Animal Health (OIE) issued Interim Guidance (56) titled, “*Reducing public health risks associated with the sale of live wild animals of mammalian species in tradition food markets.”* It called on nations to “suspend the trade in live caught wild animals of mammalian species for food or breeding purposes and close sections of food markets selling live caught wild animals of mammalian species as an emergency measure unless demonstrable effective regulations and adequate risk assessment are in place.” The guidance is focused on markets open to the public, where the conditions that provide the opportunities for animal viruses to amplify and transmit to new hosts, including humans, are clear to the observer and also documented in studies in regional markets (57). Additional surveillance for viruses of pandemic potential along wildlife supply chains, and a more comprehensive understanding of the complexity of wildlife supply chains is needed to inform wildlife trade regulation policy. In the interim, mitigation measures should consider that the wildlife trade spillover interface contains novel viruses, which may not be detectable with current diagnostic tests. Among nations, China has initiated a multi-sectoral and sustained crackdown on illegal wildlife trade and legislative reforms designed to completely phase out the farming/sourcing, trade, and consumption of terrestrial wildlife as food (58). It remains to be seen whether or not additional countries will follow this example.

## Data Availability Statement

The datasets presented in this study can be found in online repositories. The names of the repository/repositories and accession number(s) can be found in the article/Supplementary Material.

## Ethics Statement

The animal study was reviewed and approved by University of California, Davis Institutional Animal Care and Use Committee (protocol number: 16048).

## Author Contributions

NN, AL, CJ, JM, SO, and AF designed the study. NN, NLo, PN, NT, LH, PL, and AF coordinated, facilitated, and implemented field surveillance. NN, AL, LQ, and NH conducted the laboratory diagnostics and analyses. NN, HThu, NT, TP, VH, NLa, CJ, JM, CW, SO, and AF oversaw and supported project implementation. NN, AL, HThu, NA, SR, and AF collected and analyzed wildlife confiscation data. NN, AL, and AF drafted the original manuscript. HThu, NA, CJ, JM, SR, CW, and SO critically reviewed and improved the manuscript. All authors contributed to the article and approved the submitted version.

## Funding

This study was made possible by the generous support of the American people through the United States Agency for International Development (USAID) Emerging Pandemic Threats PREDICT-2 project (cooperative agreement number AID-OAA-A-14-00102). The funders had no role in study design, data collection and analysis, decision to publish, or preparation of the manuscript.

## Conflict of Interest

NN, AL, HThu, NLo, PN, NA, SR, CW, SO, and AF were employed by Wildlife Conservation Society. NT and LH was employed by Save Vietnam's Wildlife. TP and HTha were employed by Cuc Phuong National Park. NP, VH, and LQ were employed by Regional Animal Health Office No. 6. The remaining authors declare that the research was conducted in the absence of any commercial or financial relationships that could be construed as a potential conflict of interest.

## Publisher's Note

All claims expressed in this article are solely those of the authors and do not necessarily represent those of their affiliated organizations, or those of the publisher, the editors and the reviewers. Any product that may be evaluated in this article, or claim that may be made by its manufacturer, is not guaranteed or endorsed by the publisher.
