# Supplementary material for: Evidence of SARS-CoV-2 Related Coronaviruses Circulating in Sunda pangolins (Manis javanica) Confiscated From the Illegal Wildlife Trade in Viet Nam
Source: Front Public Health. 2022 Mar 9;10:826116. doi: 10.3389/fpubh.2022.826116 (PMC8959545; doi:10.3389/fpubh.2022.826116)
Supplement: Supplementary file 1 [file Table_1.DOCX]

**Supplementary Table 1**: Sarbecovirus reference and outgroup sequences included in the phylogenetic trees for this study.

| Accession number | Sequence name | Host | Country | Collection year |
| --- | --- | --- | --- | --- |
| GenBank: JX993988 | Bat CoV Cp/Yunnan2011 | *Chaerephon plicata* | China | 2011 |
| GenBank: AY278488 | SARS-CoV-1 BJ01 | *Homo sapiens* | China | 2003 |
| GenBank: KF569996 | Bat CoV LYRa11 | *Rhinolophus affinis* | China | 2011 |
| GenBank: DQ412042 | Bat CoV Rf1 | *Rhinolophus ferrumequinum* | China | 2004 |
| GenBank: KJ473813 | Bat CoV BtRf/SX2013 | *Rhinolophus ferrumequinum* | China | 2013 |
| GenBank: KP886808 | Bat CoV YNLF 31C | *Rhinolophus ferrumequinum* | China | 2013 |
| GenBank: KU182964 | Bat CoV JTMC15 | *Rhinolophus ferrumequinum* | China | 2013 |
| GenBank: DQ412043 | Bat CoV Rm1 | *Rhinolophus macrotis* | China | 2004 |
| GenBank: KF294457 | Bat CoV Longquan-140 | *Rhinolophus monoceros* | China | 2012 |
| GenBank: JX993987 | Bat CoV Rp/Shaanxi2011 | *Rhinolophus pusillus* | China | 2011 |
| GenBank: DQ022305 | Bat CoV HKU3-1 | *Rhinolophus sinicus* | China | 2004 |
| GenBank: FJ588686 | Bat CoV Rs672/2006 | *Rhinolophus sinicus* | China | 2006 |
| GenBank: KC881006 | Bat CoV Rs3367 | *Rhinolophus sinicus* | China | 2012 |
| GenBank: KJ473815 | Bat CoV GX2013 | *Rhinolophus sinicus* | China | 2013 |
| GenBank: KY417147 | Bat CoV Rs4237 | *Rhinolophus sinicus* | China | 2013 |
| GenBank: AY572038 | Civet SARS-CoV-1 civet020 | *Paguma larvata* | China | 2004 |
| GenBank: NC_045512 | SARS-CoV-2 Wuhan-Hu-1 | *Homo sapiens* | China | 2019 |
| GenBank: MT919526 | Mink SARS-CoV-2 Mink/DK/AD4_Farm1/2020 | *Neogale vison* | Denmark | 2020 |
| GISAID: EPI_ISL_410721 | Pangolin CoV 1/2019_GD | *Manis javanica* | China | 2019 |
| GenBank: MT040333 | Pangolin CoV P4L_GX | *Manis javanica* | China | 2017 |
| GenBank: MT040335 | Pangolin CoV P5L_GX | *Manis javanica* | China | 2017 |
| GenBank: MT040334 | PangolinCoV P1E_GX | *Manis javanica* | China | 2017 |
| GenBank: MT040336 | Pangolin CoV P5E_GX | *Manis javanica* | China | 2017 |
| GenBank: MT072864 | Pangolin CoV P2V_GX | *Manis javanica* | China | 2017 |
| GenBank: MT072865 | Pangolin CoV P3B_GX | *Manis javanica* | China | 2017 |
| GISAID: EPI_ISL_410544 | Pangolin CoV P2S_GD | *Manis javanica* | China | 2019 |
| GenBank: MT121216 | Pangolin CoV MP789_GD | *Manis javanica* | China | 2019 |
| GISAID: EPI_ISL_471468 | Pangolin CoV FM45_9_GD | *Manis javanica* | China | 2019 |
| GISAID: EPI_ISL_471467 | Pangolin CoV A22_2_GD | *Manis javanica* | China | 2019 |
| GISAID: EPI_ISL_471469 | Pangolin CoV SM44_9_GD | *Manis javanica* | China | 2019 |
| GISAID: EPI_ISL_471470 | Pangolin CoV SM79_9_GD | *Manis javanica* | China | 2019 |
| GISAID: EPI_ISL_610156 | Pangolin CoV MP20_YN | *Manis pentadactyla* | China | 2017 |
| GenBank: MW251308 | Bat CoV RacCS203 | *Rhinolophus acuminatus* | Thailand | 2020 |
| GenBank: MN996532 | Bat CoV RaTG13 | *Rhinolophus affinis* | China | 2013 |
| GenBank: LC556375 | BatCoV Rc-o319 | *Rhinolophus cornutus* | Japan | 2013 |
| GenBank: MW201981 | Bat CoV RmYN02 | *Rhinolophus malayanus* | China | 2019 |
| GenBank: MG772933 | Bat CoV ZC45 | *Rhinolophus pusillus* | China | 2017 |
| GenBank: MG772934 | Bat CoV ZXC21 | *Rhinolophus pusillus* | China | 2015 |
| GISAID: EPI_ISL_852604 | Bat CoV RShSTT182 | *Rhinolophus shameli* | Cambodia | 2010 |
| GISAID: EPI_ISL_852605 | Bat CoV RShSTT200 | *Rhinolophus shameli* | Cambodia | 2010 |
| GenBank: MW703458 | Bat CoV PrC31 | *Rhinolophus blythi* | China | 2018 |
| GISAID: EPI_ISL_1699446 | Bat CoV RpYN06 | *Rhinolophus pusillus* | China | 2020 |
| GenBank: MZ081380 | Bat CoV RsYN04 | *Rhinolophus stheno* | China | 2020 |
| GenBank: MZ081378 | Bat CoV RmYN08 | *Rhinolophus malayanus* | China | 2020 |
| GenBank: MZ081376 | Bat CoV RmYN05 | *Rhinolophus malayanus* | China | 2020 |
| Outgroup: |  |  |  |  |
| GenBank: KY352407 | Bat CoV BtKY72 | *Rhinolophus*sp. | Kenya | 2007 |

**Supplementary Table 2:** Acknowledgement table of GISAID data used in this study. We gratefully acknowledge the Authors from the Originating laboratories responsible for obtaining the specimens and the Submitting laboratories where genetic sequence data were generated and shared via the GISAID Initiative, on which some of our analysis are based.

| Virus name | GISAID Accession No. | Collection date | Originating laboratory | Submitting laboratory | Authors |
| --- | --- | --- | --- | --- | --- |
| hCoV-19/pangolin/Guangxi/P2S/2017 | EPI_ISL_410544 | 2017 | Beijing Institute of Microbiology and Epidemiology | Beijing Institute of Microbiology and Epidemiology | Wu-Chun Cao; Tommy Tsan-Yuk Lam; Na Jia; Ya-Wei Zhang; Jia-Fu Jiang; Bao-Gui Jiang |
| hCoV-19/pangolin/Guangdong/1/2019 | EPI_ISL_410721 | 2019 | South China Agricultural University | South China Agricultural University | Yongyi Shen, Lihua Xiao, Wu Chen |
|  | EPI_ISL_471467 | 2019 | South China Agricultural University | South China Agricultural University | Yongyi Shen, Wu Chen |
|  | EPI_ISL_471468 | 2019 | South China Agricultural University | South China Agricultural University | Yongyi Shen, Wu Chen |
|  | EPI_ISL_471469 | 2019 | South China Agricultural University | South China Agricultural University | Yongyi Shen, Wu Chen |
| hCoV-19/pangolin/Guangdong/SM79-9/2019 | EPI_ISL_471470 | 2019 | South China Agricultural University | South China Agricultural University | Yongyi Shen, Wu Chen |
| hCoV-19/pangolin/Yunnan/MP20/2017 | EPI_ISL_610156 | 2017-08-29 | State Key Laboratory of Genetic Resources and Evolution, Kunming Institute of Zoology, Chinese Academy of Sciences | State Key Laboratory of Genetic Resources and Evolution, Kunming Institute of Zoology, Chinese Academy of Sciences | Jian-Bo Li, Hang Liu, Ting-Ting Yin, Min-Sheng Peng, Ya-Ping Zhang |
| hCoV-19/bat/Cambodia/RShSTT182/2010 | EPI_ISL_852604 | 2010-12-06 | Virology Unit, Institut Pasteur du Cambodge | G5 Evolutionary Genomics of RNA viruses, Virology Department, Institut Pasteur | Vibol Hul, Deborah Delaune, Erik A Karlsson, Ou Tey Putita, Alexandre Hassanin, Artem Baidaliuk, Fabiana Gámbaro, Vuong Tan Tu, Lucy Keatts, Jonna Mazet, Christine Johnson, Philippe Buchy, Philippe Dussart, Tracey Goldstein, Etienne Simon-Lorière, Veasna Duong |
| hCoV-19/bat/Cambodia/RShSTT200/2010 | EPI_ISL_852605 | 2010-12-06 | Virology Unit, Institut Pasteur du Cambodge | G5 Evolutionary Genomics of RNA viruses, Virology Department, Institut Pasteur | Vibol Hul, Deborah Delaune, Erik A Karlsson, Ou Tey Putita, Alexandre Hassanin, Artem Baidaliuk, Fabiana Gámbaro, Vuong Tan Tu, Lucy Keatts, Jonna Mazet, Christine Johnson, Philippe Buchy, Philippe Dussart, Tracey Goldstein, Etienne Simon-Lorière, Veasna Duong |
| hCoV-19/bat/Yunnan/RpYN06/2020 | EPI_ISL_1699446 | 2020-05-25 | Shandong First Medical University & Shandong Academy of Medical Sciences | Shandong First Medical University & Shandong Academy of Medical Sciences | Weifeng Shi, Edward C. Holmes, Alice C. Hughes, Hong Zhou, Jingkai Ji, Xing Chen, Yuhai Bi, Juan Li, Tao Hu, Yanhua Chen |

**Supplementary Table 3: Results by specimen type of Sarbecovirus screening using the RT-PCR assay targeting the E gene**

| **Animal ID** | **Species name** | **Sampling date** | **Oral swab** | **Rectal swab** |
| --- | --- | --- | --- | --- |
| VNAA0204 | Sunda pangolin (*Manis javanica*) | 7/24/2018 | Positive | Positive |
| VNAA0207 | Sunda pangolin (*Manis javanica*) | 7/24/2018 | Positive | Positive |
| VNAA0208 | Sunda pangolin (*Manis javanica*) | 7/24/2018 | Negative | Positive |
| VNAA0218 | Sunda pangolin (*Manis javanica*) | 7/24/2018 | Positive | Positive |
| VNAA0219 | Sunda pangolin (*Manis javanica*) | 7/24/2018 | Positive | Positive |
| VNAA0226 | Sunda pangolin (*Manis javanica*) | 7/25/2018 | Positive | Positive |
| VNAA0227 | Sunda pangolin (*Manis javanica*) | 7/25/2018 | Negative | Positive |

**Supplementary Table 4: GenBank accession numbers of pangolin CoV sequences generated in this study**

| **Specimen ID** | **RdRp gene region 1** | **RdRp gene region 2** |
| --- | --- | --- |
| VNAA0204.RST | OK510878 | NA |
| VNAA0207.RST | OK510877 | OK510887  OK510883 |
| VNAA0208.RST | OK510879  OK510873  OK510874 | OK510888 |
| VNAA0218.RST | OK510880  OK510875  OK510876 | OK510889  OK510884 |
| VNAA0219.RST | OK510881 | OK510892  OK510885  OK510886 |
| VNAA0226.RST | NA | OK510890 |
| VNAA0227.RST | OK510882 | OK510891 |
